# Optimizing Divalent Cation Supplementation to Enhance the Production of the Kimchi Starter Strain *Latilactobacillus curvatus* WiKim0094

**DOI:** 10.4014/jmb.2505.05011

**Published:** 2025-07-18

**Authors:** Seulbi Kim, Chang Hee Jeong, Jong-Cheol Kim, Moeun Lee, Seul-Gi Jeong, Ho Myeong Kim, Hae Woong Park

**Affiliations:** 1 Technology Innovation Research Division, World Institute of Kimchi, Gwangju 61755, Republic of Korea; 2 Department of Agricultural Chemistry, Institute of Environmentally Friendly Agriculture, College of Agriculture and Life Sciences, Chonnam National University, Gwangju 61186, Republic of Korea

**Keywords:** *Latilactobacillus curvatus*, kimchi starter, divalent cation, response surface methodology, transcriptomics

## Abstract

To support the industrial-scale production of kimchi starter cultures, this study aimed to enhance the cell density of *Latilactobacillus curvatus* WiKim0094 by optimizing the composition of divalent cations in a nutrient-limited medium. Screening experiments identified Mg^2+^, Mn^2+^, and Ca^2+^ as key factors that significantly promoted cell growth. Using a Box-Behnken design, their optimal concentrations were determined to be 3.0, 0.1, and 4.4 mM, respectively, resulting in a 5.7-fold increase in the maximum viable cell count compared with the control. Comparative transcriptomic analysis revealed that supplementation with these metal ions activated pathways associated with nucleic acid synthesis, energy metabolism, nutrient transport, cell structure formation, and stress resistance. These findings provide mechanistic insights into divalent cation-mediated enhancement of microbial growth and propose a cost-effective strategy for the large-scale cultivation of kimchi starter strains. Future studies will focus on validating this approach in large-scale bioreactors to facilitate industrial application.

## Introduction

Kimchi is a traditional Korean fermented vegetable product widely recognized for its distinctive flavor and health-promoting properties [1]. These characteristics arise from interactions among various ingredients, such as kimchi cabbage, radish, chili peppers, and garlic, and a complex microbial consortium dominated by lactic acid bacteria (LAB) [2]. During fermentation, LAB become the predominant microbial population, producing organic acids that lower the pH and significantly contribute to the flavor, aroma, and microbiological safety of the final product [3].

Tailored starter cultures are increasingly employed in kimchi fermentation to enhance sensory attributes, extend shelf life, and ensure consistent quality while delivering additional health benefits [4, 5]. The global starter culture market, valued at USD 1.3 billion in 2025, is projected to expand at a compound annual growth rate (CAGR) of 6.3%, reaching USD 2.5 billion by 2035 [6]. This trend is driven by growing consumer demand for fermented foods, probiotics, and plant-based alternatives.

In large-scale kimchi manufacturing, high cell density is critical for establishing LAB dominance during fermentation, ensuring product uniformity, and achieving initial inoculum levels of at least 7–8 log CFU/g [7]. Although traditional approaches such as temperature and pH adjustments have been used to enhance LAB growth, they have inherent limitations. For instance, maintaining precise temperature control in large fermenters increases energy demands, and chemical buffering agents may leave undesirable residues in the final product [8].

To overcome these challenges, recent efforts have focused on optimizing the composition of culture media, particularly with respect to essential trace elements [9-11]. Among these, divalent metal ions, including Mg^2+^, Mn^2+^, Ca^2+^, Cu^2+^, Zn^2+^, and Fe^2+^, are known to play vital roles in enzymatic catalysis, membrane integrity, and various metabolic processes [12-14]. Due to their complex and species-specific interactions, single-factor optimization is insufficient for determining ideal supplementation levels. Accordingly, statistical approaches such as response surface methodology (RSM), particularly the Box-Behnken Design (BBD), have gained prominence in systematically optimizing ion concentrations to maximize microbial proliferation [15, 16].

*L. curvatus* WiKim0094, originally isolated from kimchi, has recently garnered attention as a promising next-generation starter culture due to its stable predominance and adaptability during fermentation. Notably, its cell-free supernatant has demonstrated potential for mitigating neuroinflammation in lipopolysaccharide-induced glial cell models by suppressing nitric oxide production and attenuating reactive gliosis [17]. Based on these favorable characteristics, the present study aimed to enhance the growth of WiKim0094 by refining the trace metal composition of a diluted MRS-based medium, thereby developing a more efficient and scalable cultivation strategy. Initially, six divalent cations (Mg^2+^, Mn^2+^, Cu^2+^, Ca^2+^, Zn^2+^, and Fe^2+^) were evaluated in single-factor experiments to assess their impact on bacterial growth. The three most effective ions (Mg^2+^, Mn^2+^, and Ca^2+^) were subsequently optimized using the BBD to maximize viable cell density. Finally, comparative transcriptomic analyses were performed between the optimized metal-supplemented medium (MMC) and the control to elucidate the molecular mechanisms underlying metal ion–driven growth enhancement.

This study contributes by (i) identifying and optimizing trace metal ions that significantly promote the growth of kimchi-derived LAB, (ii) validating the optimized conditions using multifactorial statistical methods, and (iii) providing transcriptomic insights into how divalent metal ions modulate microbial metabolism and regulatory networks. These findings are expected to support the development of scalable, cost-effective processes for kimchi starter culture production and to advance our understanding of the critical roles of metal ions in microbial physiology.

## Materials and Methods

### Bacterial Strain and Culture Conditions

WiKim0094 (accession number: PRJNA845009), originally isolated from kimchi, was pre-cultured in MRS broth (BD-Difco, USA) at 30°C for 24 h. Following pre-cultivation, cells were harvested by centrifugation at 12,000 ×*g* for 5 min at 4°C, washed three times with 0.85% (w/v) saline, and resuspended. The washed cells were inoculated at a final concentration of 10^7^ colony-forming units (CFU)/ml into diluted MRS broth and incubated at 30°C for 24 h. A 5-fold diluted MRS (1/5 MRS) broth was used as a minimal medium to simulate a nutrient-limited environment conducive to assessing metabolic responses in WiKim0094.

### Divalent Cation Screening by Single-Factor Test

To evaluate the effects of divalent cations on the growth of WiKim0094, a single-factor analysis was performed using six ions (Mg^2+^, Mn^2+^, Cu^2+^, Ca^2+^, Zn^2+^, and Fe^2+^). Concentration ranges for each ion were selected based on the metal ion content in kimchi as reported by [18], and are summarized in Table 1. As described above WiKim0094 cells were inoculated into 1/5 MRS broth supplemented with low, medium, and high concentrations of each divalent cation. Stock solutions were prepared at 100-fold higher concentrations, sterilized through 0.2 μm membrane filters, and added to the medium prior to inoculation. Cultures were incubated statically at 30°C for 24 h. Following incubation, serial dilutions were plated on MRS agar to determine CFU. All experiments were performed in triplicate.

### Optimization of Divalent Cation Concentrations Using Box-Behnken Design

To determine the optimal concentrations of MgSO_4_ (X_1_), MnSO_4_ (X_2_), and CaCl_2_ (X_3_) for promoting the growth of WiKim0094, response surface methodology based on BBD was employed. These three cations were selected based on results from the single-factor test. Each variable was evaluated at three coded levels (–1, 0, +1), as shown in Table 2. Concentration ranges were determined based on the highest levels that did not induce precipitation in preliminary experiments (data not shown). The experimental matrix for BBD is presented in Table 3. Statistical analysis and response optimization were conducted using Minitab version 18 (Minitab Inc., USA). The effect of each variable on CFU was modeled using the following second-order polynomial Eq. (1):

$$y=b_{0}+\sum b_{i}x_{i}+\sum b_{ii}x_{i}^{2}+\sum b_{ij}x_{i}x_{j}+e\left(i,=1,\ldots ,k,j=i+1\right)$$ (1)

where *y* is the predicted response, *b_0_* is the constant, and *b_i_*, *b_ii_*, and *b_ij_* are the coefficients for linear, quadratic, and interaction effects, respectively. *x_i_* and *x_j_* are the coded independent variables, *k* is the number of variables, and *e* is the error term.

### RNA Isolation, cDNA Library Construction, and RNA Sequencing

WiKim0094 was cultured in 1/5 MRS broth supplemented with 3.0 mM MgSO_4_, 0.1 mM MnSO_4_, and 4.4 mM CaCl_2_ (hereafter referred to as MMC medium). Following the procedure described in Section 2.1, cells were harvested after 12 h and 24 h of incubation. Total RNA was extracted using the RNeasy Plus Mini Kit (Qiagen, Germany) according to the manufacturer's instructions. RNA isolation was performed in triplicate for both the treatment group (MMC medium) and the control group (1/5 MRS medium without metal ion supplementation).

RNA concentration and purity were assessed using a NanoDrop One spectrophotometer (Thermo Fisher Scientific, USA). Samples with OD_260/280_ values between 1.8 and 2.2, RNA concentrations ≥100 ng/μl, and total yields >2 μg were deemed suitable for downstream applications. Ribosomal RNA was depleted using the NEBNext rRNA Depletion Kit (New England Biolabs Inc., USA), and cDNA libraries were prepared using the TruSeq Stranded Total RNA Sample Prep Kit with Ribo-Zero H/M/R (Illumina, USA). Amplified cDNA fragments were subjected to paired-end sequencing (150 bp read length) on the Illumina NovaSeq X Plus platform. Sequencing reads were aligned to the reference genome of WiKim0094 and normalized using CLRNASeq™ software (Chunlab, Republic of Korea).

### Analysis of Differentially Expressed Genes and Pathway Enrichment

The expression levels of genes were normalized using the trimmed mean of M-values (TMM). To analyze the gene expression profile of WiKim0094 in response to metal ion supplementation, samples were collected at 12 h and 24 h after inoculation, based on growth curve analysis. Sample groups were designated as follows: NC12 and NC24 (negative control at 12 h and 24 h, respectively), and T12 and T24 (MMC-treated samples at 12 h and 24 h, respectively). Differentially expressed genes (DEGs) were identified for T12 vs. NC12 and T24 vs. NC24 comparisons, using a threshold of |log_2_ fold change| > 1 and *p*-value < 0.05. Fold changes were calculated as the ratio of expression level in treated samples relative to controls. Statistical significance was assessed using *edgeR*. Gene Ontology (GO) enrichment analysis and classification by the Evolutionary Genealogy of Genes: Non-supervised Orthologous Groups (EggNOG) database were performed using CLRNASeq software.

### Quantitative Real-Time Polymerase Chain Reaction (qRT-PCR)

The expression of selected genes was validated by qRT-PCR using the CFX96 Real-Time PCR Detection System (Bio-Rad Laboratories, USA) with AMPIGENE qPCR Green Mix Hi-ROX (Enzo Life Science, USA) containing SYBR Green dye. The thermal cycling conditions were as follows: initial denaturation at 95°C for 2 min, followed by 40 cycles of 95°C for 5 sec and 56°C for 20 sec. Primer sequences are listed in Table S1. All reactions were conducted in triplicate using the same RNA preparations employed in the RNA-seq analysis. Gene expression levels were normalized to the reference genes *recA*, *gyrB*, and *rpoB*, and relative fold changes were calculated using the 2^–ΔΔCt^ method.

### Statistical Analysis

All experiments were performed in triplicate, and data are presented as mean ± standard deviation. Statistical significance was determined using one-way analysis of variance (ANOVA), followed by Tukey’s honest significant difference (HSD) test. A *p*-value < 0.05 was considered statistically significant. All analyses were performed using SPSS software (version 20; SPSS Inc., USA).

## Results and Discussion

### Effect of Divalent Cations on the Growth of WiKim0094

**Fig. 1:**
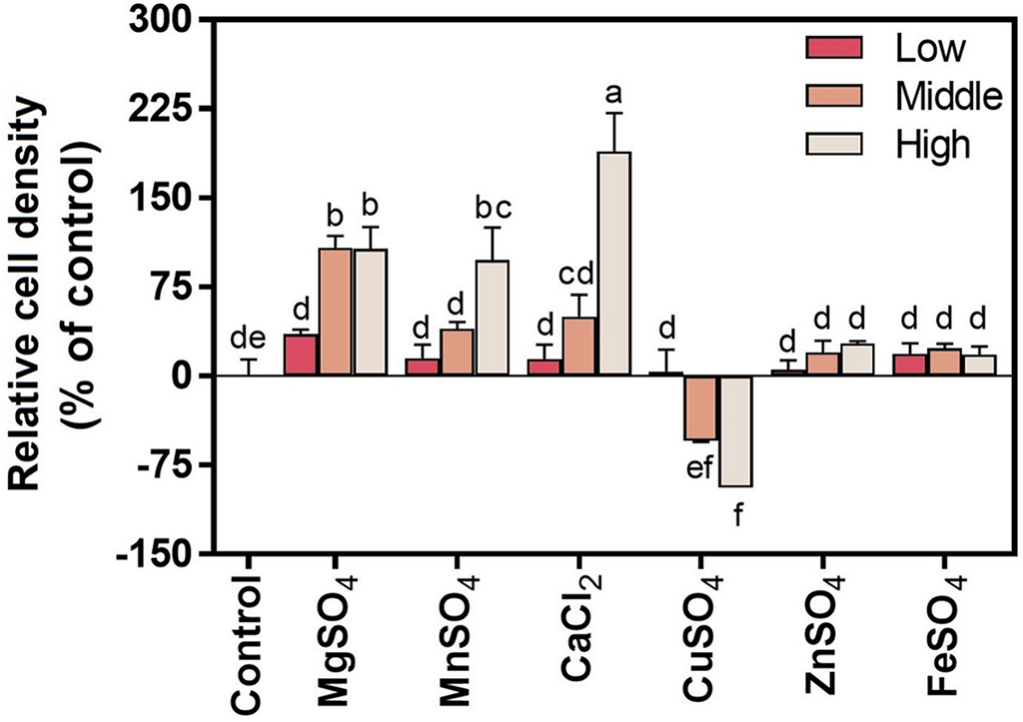
Effect of different divalent cations (MgSO_4_, MnSO_4_, CaCl_2_, CuSO_4_, ZnSO_4_, and FeSO_4_) at low, medium, and high concentrations on the relative viability of *L. curvatus* WiKim0094 compared with the control. Colony-forming units after 24 h of incubation are expressed as a percentage of the control (1/5 MRS broth without added cations). Treatment concentrations were as follows: MgSO_4_, 1, 10, and 20 mM; MnSO_4_, 0.1, 1, and 5 mM; CaCl_2_, 0.1, 1, and 10 mM; CuSO_4_, 0.01, 0.1, and 1 mM; ZnSO_4_, 0.01, 0.1, and 1 mM; FeSO_4_, 0.005, 0.05, and 0.5 mM. Bars represent the mean ± standard deviation. Different letters indicate statistically significant differences among treatments (*p* < 0.05).

To ensure food safety in kimchi fermentation, six GRAS-designated divalent cations (Mg^2+^, Mn^2+^, Ca^2+^, Zn^2+^, Cu^2+^, and Fe^2+^) were selected based on their physiological relevance to lactic acid bacteria [19]. The growth of WiKim0094 was evaluated after 24 h of incubation in the presence of six different divalent metal ions (Fig. 1), each of which exerted distinct effects on bacterial proliferation. Among them, Mg^2+^, Mn^2+^, and Ca^2+^ significantly enhanced growth in a dose-dependent manner. Ca^2+^ exhibited the most pronounced effect, with a concentration of 10 mM resulting in a 189.1% increase in CFU relative to the untreated control. These findings are consistent with prior studies reporting that Mg^2+^, Mn^2+^, and Ca^2+^ generally promote microbial growth and metabolic activity [20, 21]. For example, Mg^2+^ has been shown to extend the exponential growth phase and reduce protein acetylation in microorganisms such as *Lactobacillus bifermentans*, *Escherichia coli*, *Vibrio fischeri*, and *Bacillus subtilis* [22, 23]. Likewise, Mn^2+^ supplementation has been reported to enhance cell density in *Lactiplantibacillus plantarum* WCFS1 by approximately 3-fold compared with untreated controls [24]. Ca^2+^ has also been identified as a critical factor in optimizing the growth of LAB, including *L. plantarum* B1.3 [25].

In this study, the pronounced growth-promoting effect of Ca^2+^ suggests that WiKim0094 may possess a high affinity or specific requirement for calcium to support key metabolic processes. This observation aligns with the established roles of Ca^2+^ in stabilizing bacterial membranes and mediating intracellular signaling [26, 27]. The contributions of Mg^2+^ and Mn^2+^ to WiKim0094 growth are likely attributable to their known involvement as enzymatic cofactors and in oxidative stress defense mechanisms [28].

In contrast, Cu^2+^ exerted a growth-inhibitory effect, with higher concentrations leading to a marked reduction in viable cell counts. This observation is consistent with prior research indicating that Cu^2+^ can delay microbial growth by inhibiting the biosynthesis of primary metabolites essential for cell development and replication [11]. The intracellular requirement for Cu^2+^ is generally low (1–10 μM), and many anaerobic bacteria have evolved reduced dependence on this metal [29]. The observed inhibitory effect of Cu^2+^ on WiKim0094 underscores the need for its careful regulation or exclusion from media formulations intended for large-scale cultivation.

Zn^2+^ and Fe^2+^ produced modest increases in CFU, ranging from 6.0% to 27.9% and 18.4% to 23.9%, respectively; however, these changes were not statistically significant. This limited effect may be attributed to previous reports showing that intracellular Zn^2+^ concentrations can vary widely across microbial species [29], and that Fe^2+^ requirements are typically minimal in certain LAB strains [30-32]. For instance, while Zn^2+^ concentrations exceeding 619 μM support maximum growth in *Lactobacillus thermophilus*, elevated levels may inhibit other LAB species, including *L. acidophilus* and *L. casei* [33]. Similarly, although Fe^2+^ is essential for DNA-binding stabilization in *B. subtilis*, its intracellular free concentration is typically maintained below 1 μM [34].

Collectively, these findings highlight the pivotal role of divalent cations in modulating the growth dynamics of WiKim0094. The identification of Mg^2+^, Mn^2+^, and Ca^2+^ as primary growth-promoting factors, along with the inhibitory effect of Cu^2+^, provides mechanistic insight into ion-specific responses and informs the development of optimized media formulations for industrial-scale cultivation of kimchi starter cultures. While targeted supplementation with select metal ions may enhance cell density and reduce production costs, it is essential to consider potential interactions among ions, as excessive or imbalanced combinations may exert antagonistic effects or disrupt microbial community composition.

### Optimization of Cation Concentration Using Box-Behnken Design for Enhanced WiKim0094 Growth

**Analysis of key parameters influencing WiKim0094 growth.** Based on the results of the single-factor test, the effects of three significant variables, MgSO_4_, MnSO_4_, and CaCl_2_, on the growth of *L. curvatus* WiKim0094 were investigated using BBD. The viable cell counts obtained after 24 h of incubation for each experimental run are presented in Table 3. Regression analysis was performed using Minitab software to derive a second-order polynomial equation describing the relationship between the coded variable values and CFU (Eq. (2)):

$$Y=-0.697+2.599X_{1}+1.67X_{2}+0.910X_{3}-0.1932X_{1}^{2}+0.101X_{2}^{2}-0.0356X_{3}^{2}-0.971X_{1}X_{2}-0.1975X_{1}X_{3}-0.019X_{2}X_{3}$$ (2)

This model was used to predict the CFU for each experimental condition (Table 3). Fig. S1 demonstrates the correlation between the experimental and predicted CFU values. The linear regression equation (*y* = 0.9754*x* + 0.1081) and the coefficient of determination (*R*² = 0.9751) indicated a strong correlation, confirming the model’s predictive reliability.

ANOVA was conducted to evaluate the contribution of each variable to CFU (Table 4). The BBD model demonstrated high significance and prediction (*p* = 0.002, *p* < 0.01), with *R*² = 0.9751 and adjusted *R*² = 0.9302. The *p*-value for lack of fit was 0.241 (*p* > 0.05), indicating that the model adequately fit the experimental data.

Among the regression terms, the linear effects of MgSO_4_ (X_1_) and CaCl_2_ (X_3_), and the interaction effects of X_1_X_2_ and X_2_X_3_, had statistically significant influences on WiKim0094 growth (*p* < 0.05). Notably, MgSO_4_ and CaCl_2_ exhibited the strongest effects, with regression coefficients of 2.599 and 0.910, respectively (*p* < 0.0001), indicating a positive and proportional relationship with CFU. In contrast, the quadratic terms (X_1_², X_2_², and X_3_²) were not statistically significant (Fig. S2).

**Fig. 2:**
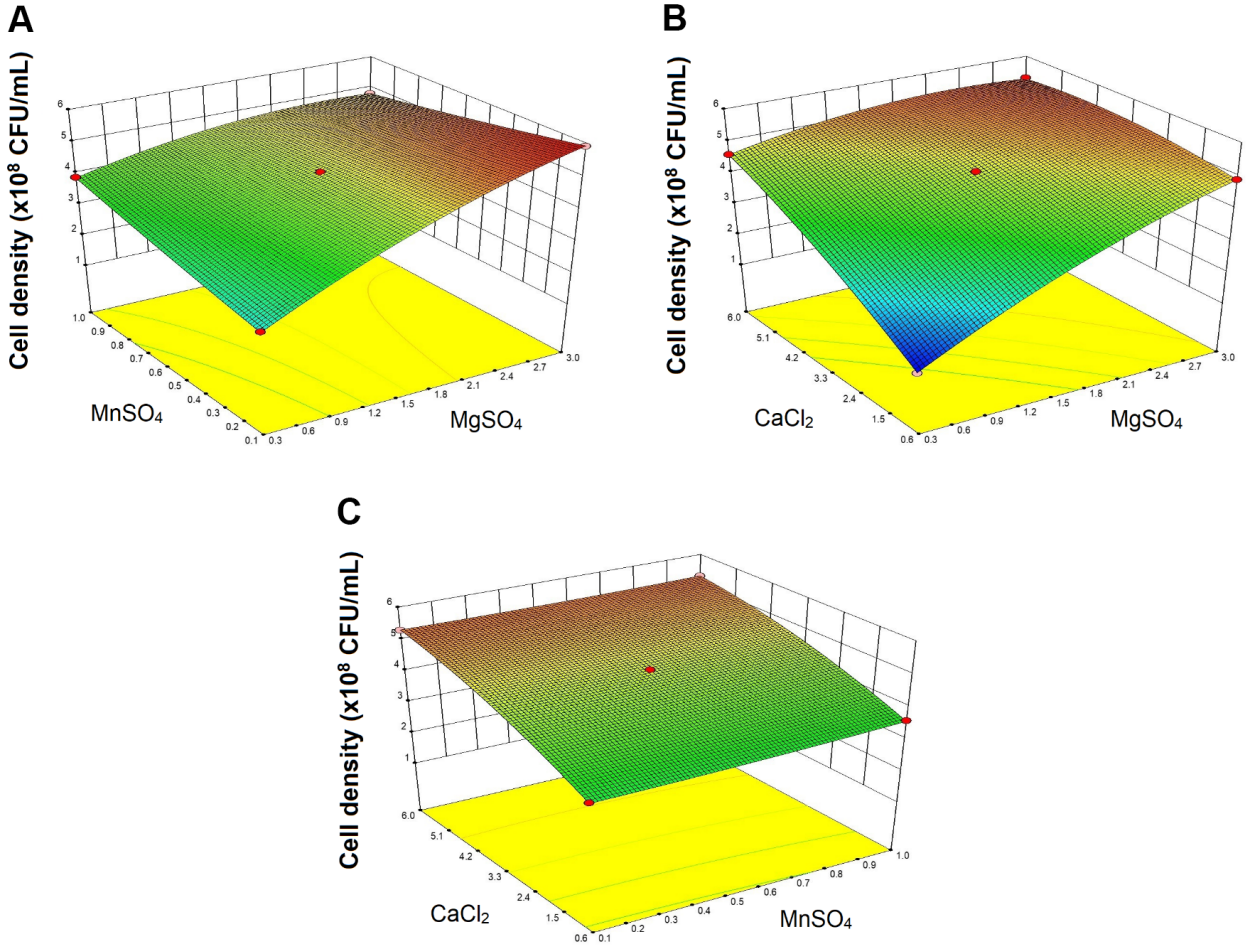
Three-dimensional response surface plots from the Box-Behnken design showing the interactive effects of divalent cations on the growth of *L. curvatus* WiKim0094. The plots show the interactions between colony-forming units (CFU) and concentrations of (**A**) MgSO_4_ and CaCl_2_, (**B**) MgSO_4_ and MnSO_4_, and (**C**) MnSO_4_ and CaCl_2_.

**Fig. 3:**
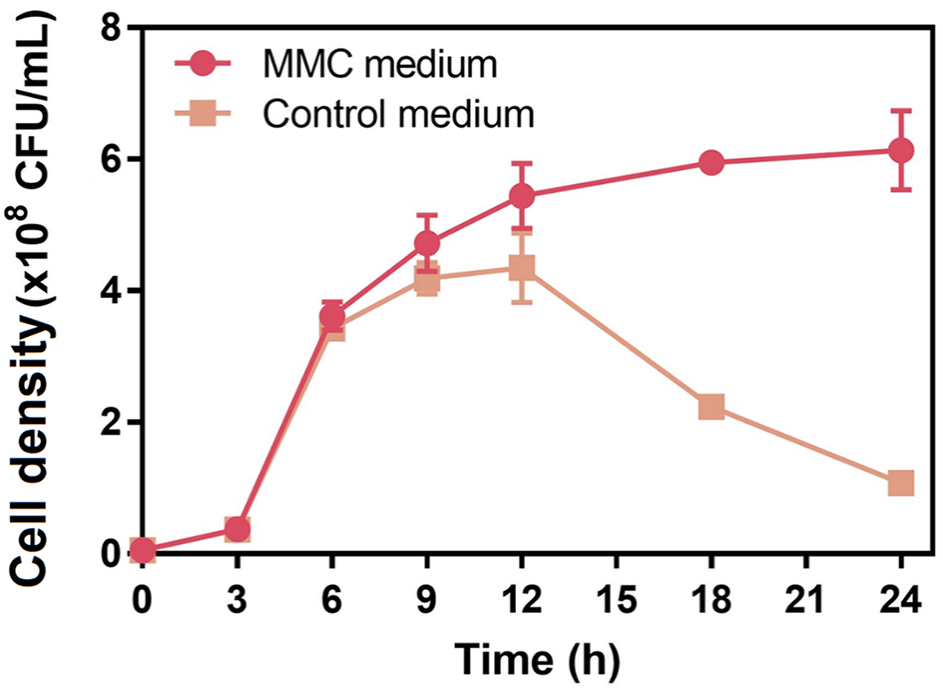
Growth curve of *L. curvatus* WiKim0094 in optimized MMC medium (●, supplemented with 3.0 mM MgSO_4_, 0.1 mM MnSO_4_, and 4.4 mM CaCl_2_) and control medium (■, 1/5 MRS broth). Viable cell counts were measured every 3 h over a 24 h incubation period. Error bars represent standard deviations from triplicate experiments.

**Optimization of parameters for enhancing the growth of WiKim0094.** Optimal concentrations for maximizing WiKim0094 growth were identified using the BBD model (Fig. 2). Under optimized conditions, 3.0 mM MgSO_4_, 0.1 mM MnSO_4_, and 4.4 mM CaCl_2_, the predicted viable cell density was 5.9 × 10^8^ CFU/ml (Fig. 3). Validation experiments conducted in triplicate under these conditions showed an average viable cell density of 6.1 × 10^8^ CFU/ml, representing a 5.7-fold increase relative to the control (1.1 × 10^8^ CFU/ml). The experimental value was approximately 104% of the predicted value and within the model’s confidence interval, confirming the robustness of the regression model.

These findings demonstrate the efficacy of divalent cation optimization in substantially enhancing the growth of WiKim0094. The strong correlation between predicted and observed outcomes supports the reliability of response surface methodology using BBD as a predictive and optimization tool for microbial culture conditions. Among the tested variables, Ca^2+^ exerted the most pronounced effect on cell proliferation, consistent with previous studies highlighting its essential roles in cellular stabilization and metabolic regulation [20, 31].

The observed increase in viable cell count suggests a synergistic effect among Mg^2+^, Mn^2+^, and Ca^2+^ in promoting critical cellular functions such as enzymatic activity, membrane integrity, and stress tolerance. The 5.7-fold enhancement in CFU underscores the potential of targeted metal ion supplementation as a cost-effective and scalable strategy for the industrial cultivation of WiKim0094.

### Functional Annotation, Pathway Enrichment, and Expression Analysis of DEGs

**Fig. 4:**
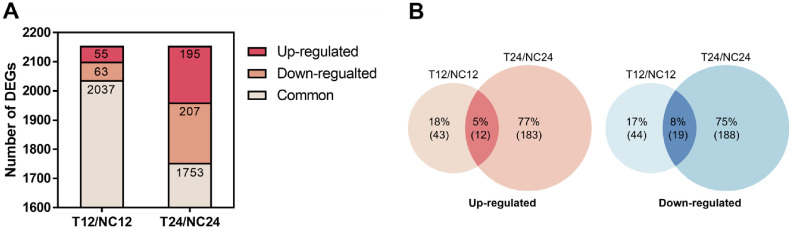
Differential gene expression in *L. curvatus* WiKim0094 under divalent cation supplementation. (**A**) Bar plot showing the number of differentially expressed genes (DEGs) in the T12/NC12 and T24/NC24 comparisons, categorized as upregulated, downregulated, or shared. (**B**) Venn diagram illustrating overlap between upregulated and downregulated DEGs across the two comparisons. T12 and T24, cultures supplemented with 3.0 mM Mg^2+^, 0.1 mM Mn^2+^, and 4.4 mM Ca^2+^ for 12 h and 24 h; NC12 and NC24, unsupplemented control cultures at the corresponding time points.

Transcriptomic analysis was performed to examine the gene expression changes in WiKim0094 cultured in minimal MRS supplemented with divalent cations (MMC medium), with the aim of identifying DEGs and elucidating their functional relevance. A total of 118 DEGs were identified in the T12/NC12 comparison group and 402 DEGs in the T24/NC24 group, using the thresholds of |log_2_ fold change| > 1 and adjusted *p* < 0.05. Among these, 55 genes were upregulated and 63 were downregulated in the T12/NC12 group, whereas 195 genes were upregulated and 207 were downregulated in the T24/NC24 group (Fig. 4A). A Venn diagram revealed both shared and unique DEGs between the two time points, highlighting the dynamic transcriptional response of WiKim0094 (Fig. 4B).

Functional annotation and enrichment analyses of DEGs were conducted using GO and EggNOG databases to determine their biological roles. Consistent with previous studies, transcriptional responses associated with Mg^2+^, Mn^2+^, and Ca^2+^ supplementation included the upregulation of genes involved in DNA and RNA biosynthesis, energy metabolism, nutrient transport, cell structure formation, and adaptation to environmental stress [27, 35, 36].

**Fig. 5:**
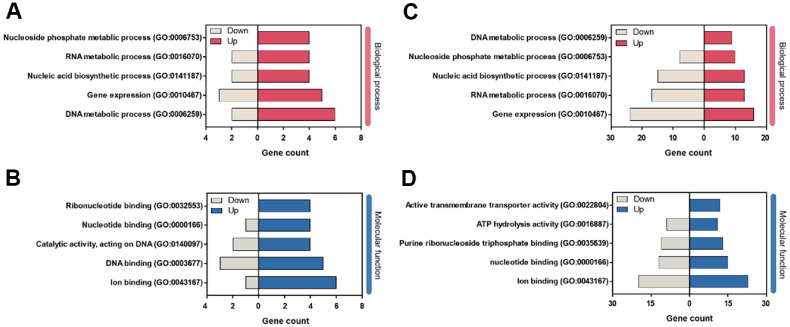
Gene ontology (GO) enrichment analysis of differentially expressed genes (DEGs) in *L. curvatus* WiKim0094 under divalent cation supplementation. (**A, C**) Enriched biological process terms for upregulated and downregulated DEGs in the T12/NC12 and T24/NC24 groups, respectively. (**B, D**) Enriched molecular function terms for upregulated and downregulated DEGs in the T12/NC12 and T24/NC24 groups, respectively. T12 and T24, cultures supplemented with 3.0 mM Mg^2+^, 0.1 mM Mn^2+^, and 4.4 mM Ca^2+^ for 12 h and 24 h; NC12 and NC24, unsupplemented control cultures at the corresponding time points.

GO enrichment analysis classified DEGs into three major categories: biological process (BP), molecular function (MF), and cellular component (CC). The BP and MF categories contained the most abundant DEGs, particularly in the T24/NC24 group. Significantly enriched BP terms included DNA metabolic process (GO:0006259), nucleic acid biosynthetic process (GO:0141187), and RNA metabolic process (GO:0016070) (Fig. 5A and 5C), indicating elevated transcriptional and translational activity under metal ion exposure [37, 38]. Within the MF category, enrichment was observed in nucleotide binding (GO:0000166), active transmembrane transporter activity (GO:0022804), and ATP hydrolysis activity (GO:0016887) (Fig. 5B and 5D). These molecular functions are likely to contribute to growth enhancement by facilitating efficient nutrient transport across the cell membrane and ensuring sufficient energy supply for protein synthesis, cell division, and activation of metabolic pathways [37].

**Fig. 6:**
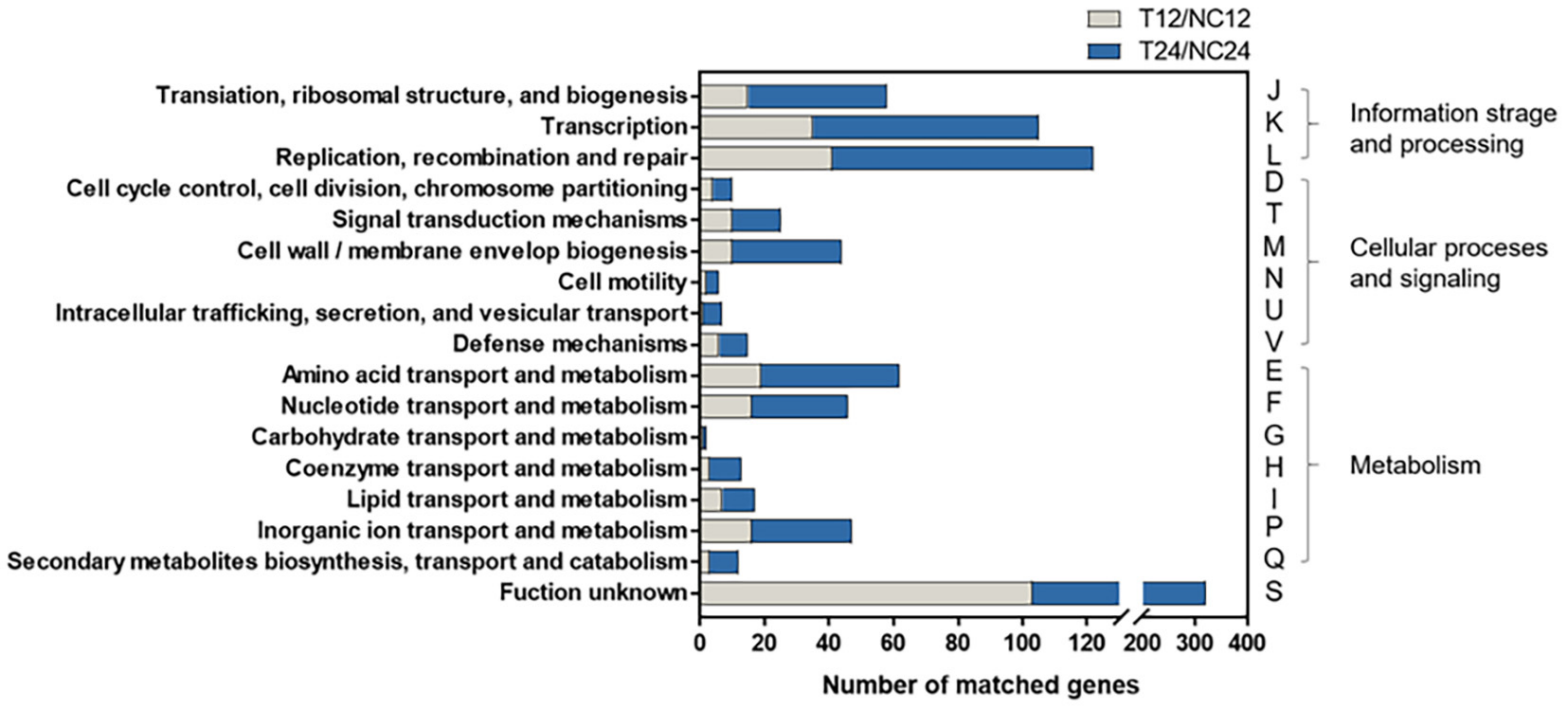
Functional annotation of differentially expressed genes (DEGs) in *L. curvatus* WiKim0094 based on EggNOG classifications under divalent cation supplementation. DEGs from T12/NC12 (gray bars) and T24/NC24 (blue bars) comparisons were grouped into three major categories: information storage and processing, cellular processes and signaling, and metabolism. T12 and T24, cultures supplemented with 3.0 mM Mg^2+^, 0.1 mM Mn^2+^, and 4.4 mM Ca^2+^ for 12 h and 24 h; NC12 and NC24, unsupplemented control cultures at the corresponding time points.

Further annotation using the EggNOG database revealed significant enrichment in functional categories related to information storage and processing (J: translation; K: transcription; L: replication, recombination, and repair), cellular processes and signaling (M: cell wall/membrane/envelope biogenesis), and metabolism (E: amino acid metabolism; F: nucleotide metabolism; P: inorganic ion transport and metabolism) (Fig. 6).

These enriched pathways reflect a coordinated transcriptional response facilitating cellular proliferation and biosynthetic activity under optimized growth conditions. Notably, enrichment of category M suggests that divalent cations contribute to cell envelope remodeling and stabilization, critical for maintaining cellular integrity during rapid growth [39, 40]. Genes involved in amino acid and nucleotide metabolism (categories E and F) were upregulated, supporting the hypothesis that biosynthetic and transport pathways are activated to meet the increased demand for macromolecule synthesis. The enrichment of inorganic ion transport and metabolism (category P) may reflect homeostatic adaptation to elevated extracellular ion concentrations, potentially mediated by regulatory feedback mechanisms.

Specific gene responses to metal ion exposure were also observed. WK_01202 (encoding a manganese ABC transporter) and WK_01814 (encoding a calcium-transporting ATPase) were both upregulated, suggesting enhanced ion uptake (Table 5) [35, 41]. Genes associated with Mg^2+^-dependent processes, including *nrdA* and *nrdB* (encoding ribonucleotide-diphosphate reductases), *purD* (encoding phosphoribosylamine-glycine ligase), and WK_00665 (encoding RNA polymerase), were also upregulated, indicating increased nucleic acid biosynthesis [42-44]. WK_00489 (encoding a UvrABC DNA repair protein) and WK_01134 (encoding ATP synthase) were upregulated as well, supporting enhanced DNA repair and ATP production under Mg^2+^ exposure [45, 46]. These findings align with previous studies showing that Mg^2+^ contributes to bacterial survival by stabilizing ribosomes, neutralizing nucleic acid charges, enhancing enzymatic activity, and promoting DNA and protein synthesis [21, 29, 47, 48].

In addition to its role in nucleic acid and energy metabolism, Mg^2+^ was found to act synergistically with Ca^2+^ in promoting lipoteichoic acid biosynthesis through activation of diacylglycerol kinase (*dgkA*), while stabilizing membrane integrity via charge neutralization of membrane lipids [28, 36, 40, 49, 50]. Upregulation of *dgkA* was observed along with other genes involved in membrane and cell wall biosynthesis, including *yoaK*, WK_01655 (membrane protein), WK_00373 (ABC transporter G family), and WK_01174 (LytR-CpsA-Psr family protein)[51-53].

Mn^2+^ was identified as a key cofactor for enzymatic reactions and oxidative stress regulation [24, 54]. Upregulation of WK_01557 (encoding thioredoxin) under Mn^2+^ exposure suggests enhanced oxidative stress resilience and cellular viability [55]. Ca^2+^ supplementation also upregulated WK_01814 (encoding an EpsG family protein), indicating increased exopolysaccharide (EPS) biosynthesis [56]. EPS production promotes acid tolerance, oxidative stress resistance, and biofilm formation, collectively enhancing bacterial survival and alleviating end-product inhibition caused by organic acid accumulation [57-60].

### Validation of DEGs by qRT-PCR

**Fig. 7:**
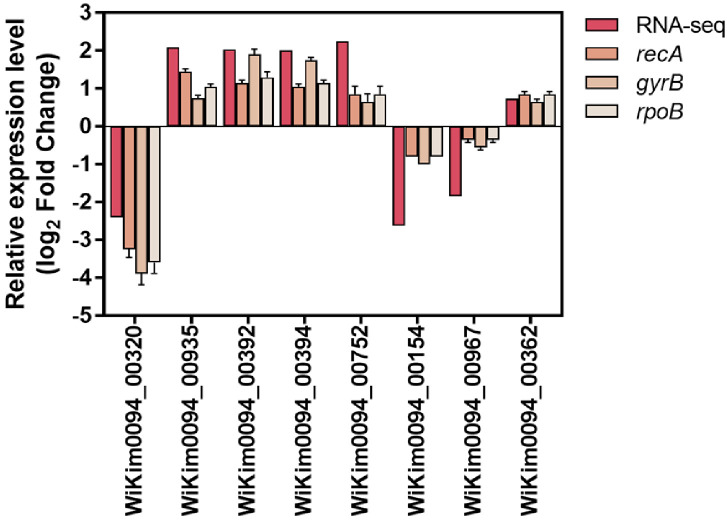
Validation of RNA-sequencing (RNA-seq) data by quantitative real-time PCR (qRT-PCR) analysis of eight selected genes in *L. curvatus* WiKim0094. The *recA*, *gyrB*, and *rpoB* genes were used as internal reference genes. Error bars represent standard deviations from three biological replicates.

Eight genes were randomly selected for validation of the RNA sequencing results using qRT-PCR. As shown in Fig. 7, the qRT-PCR results were highly consistent with the transcriptomic data. The housekeeping genes *recA*, *gyrB*, and *rpoB* exhibited *R*² of 0.79, 0.74, and 0.74, respectively, with corresponding *p*-values of 0.003, 0.006, and 0.005, indicating strong correlation.

The random selection of validation targets, along with the consistent results obtained using three independent internal controls (*recA*, *gyrB*, and *rpoB*), further underscores the reliability of the qRT-PCR analysis. Moreover, the consistency between qRT-PCR and RNA-seq data—observed across both upregulated and downregulated genes—demonstrates the technical robustness of the transcriptomic dataset and indicates unbiased quantification [61]. Collectively, these results confirm that the transcriptomic data accurately reflect the transcriptional responses of WiKim0094 under the tested conditions.

## Conclusion

This study identified metal ion compositions that enhance the cell density of *L. curvatus* WiKim0094, a kimchi-derived starter strain, and elucidated the molecular mechanisms underlying metal ion-mediated growth promotion. Optimal growth was achieved in a medium supplemented with 3.0 mM MgSO_4_, 0.1 mM MnSO_4_, and 4.4 mM CaCl_2_. Transcriptomic profiling revealed activation of pathways involved in DNA/RNA biosynthesis, energy metabolism, nutrient transport, cell structure formation, and environmental adaptation. These findings support the hypothesis that targeted metal ion supplementation promotes cellular proliferation by stabilizing enzymatic cofactors, enhancing transcriptional activity, and reinforcing membrane integrity. This culture strategy represents a promising approach for the large-scale cultivation of starter strains in the fermented food industry. Future studies should focus on optimizing scale-up processes, including bioreactor parameter adjustment and cost-benefit analysis, to facilitate the industrial application of this strategy.

## Supplemental Materials

Supplementary data for this paper are available on-line only at http://jmb.or.kr.



## Figures and Tables

**Table 1:** Concentrations of divalent cations supplemented in a 5-fold diluted MRS medium.

Metal	Cation form	Concentration (mM)		
		Low	Middle	High
Mg	MgSO_4_	1	10	20
Mn	MnSO_4_·H_2_O	0.1	1	5
Ca	CaCl_2_·2H_2_O	0.1	1	10
Cu	CuSO_4_	0.01	0.1	1
Zn	ZnSO_4_·7H_2_O	0.01	0.1	1
Fe	FeSO_4_·7H_2_O	0.005	0.05	0.5

**Table 2:** Variables and levels in the Box-Behnken design.

Independent variables	Code	Levels		
		–1	0	+1
MgSO_4_ (mM)	X_1_	0.3	1.7	3.0
MnSO_4_ (mM)	X_2_	0.1	0.6	1.0
CaCl_2_ (mM)	X_3_	0.6	3.3	6.0

**Table 3:** Three-variable Box-Behnken design for enhancing the viable cell density of *Latilactobacillus curvatus* WiKim0094.

Run	Independent variables			Viable cell density (×10^8^ CFU/ml)		
	X_1_*	X_2_**	X_3_***	Measured	Predicted
1	0	+1	+1	5.5	5.3
2	+1	–1	0	5.8	5.9
3	0	0	0	4.8	4.6
4	0	–1	–1	3.2	3.4
5	+1	0	–1	5.2	4.9
6	0	–1	+1	5.5	5.3
7	–1	0	–1	1.4	1.3
8	0	0	0	4.4	4.6
9	0	+1	–1	3.4	3.6
10	–1	0	+1	4.3	4.6
11	+1	+1	0	4.7	4.8
12	+1	0	+1	5.2	5.3
13	–1	–1	0	2.7	2.6
14	–1	+1	0	4.0	3.9
15	0	0	0	4.7	4.6

*X_1_, magnesium sulfate concentration (mM).

**X_2_, manganese concentration (mM).

***X_3_, calcium chloride concentration (mM).

**Table 4:** Analysis of variance results for Box-Behnken design.

Source	Sum of squares	Degree of freedom	Mean square	*F* value	*P* value
Model	19.6204	9	2.18005	21.74	0.002
X_1_, MgSO_4_	8.7781	1	8.77805	87.55	0.000
X_2_, MnSO_4_	0.0210	1	0.02101	0.21	0.666
X_3_, CaCl_2_	6.6795	1	6.67951	66.62	0.000
X_1_X_1_	0.4577	1	0.45771	4.56	0.086
X_2_X_2_	0.0015	1	0.00154	0.02	0.906
X_3_X_3_	0.2488	1	0.24880	2.48	0.176
X_1_X_2_	1.3924	1	1.39240	13.89	0.014
X_1_X_3_	2.0736	1	2.07360	20.68	0.006
X_2_X_3_	0.0020	1	0.00202	0.02	0.893
Residual	0.5013	5	0.10027		
Lack of Fit	0.4173	3	0.13909	3.31	0.241
Pure Error	0.0841	2	0.04203		
Total	20.1218	14			
R²	0.9751				
Adjusted R²	0.9302				

**Table 5:** Genes and proteins associated with the enhanced growth of *Latilactobacillus curvatus* WiKim0094.

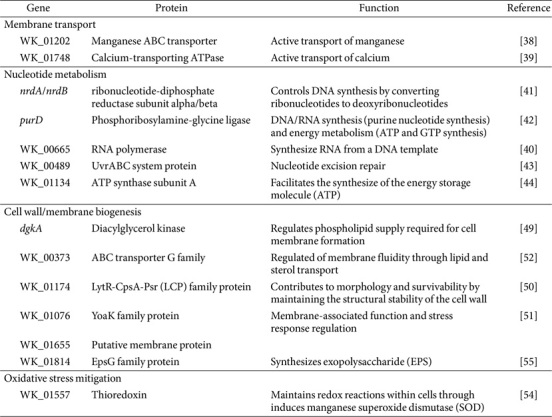

## References

[1] Cha J, Kim YB, Park SE, Lee SH, Roh SW, Son HS (2024). Does kimchi deserve the status of a probiotic food?. Crit. Rev. Food Sci. Nutr..

[2] Song HS, Lee SH, Ahn SW, Kim JY, Rhee JK, Roh SW (2021). Effects of the main ingredients of the fermented food, kimchi, on bacterial composition and metabolite profile. Food Res. Int..

[3] Kang JY, Lee M, Song JH, Choi EJ, Mun SY, Kim D (2024). Organic acid type in kimchi is a key factor for determining kimchi starters for kimchi fermentation control. Heliyon.

[4] Kim SJ, Ha S, Dang YM, Chang JY, Mun SY, Ha JH (2023). Combined non-thermal microbial inactivation techniques to enhance the effectiveness of starter cultures for kimchi fermentation. J. Microbiol. Biotechnol..

[5] Lee KW, Kim GS, Baek AH, Hwang HS, Kwon DY, Kim SG (2020). Isolation and characterization of kimchi starters *Leuconostoc mesenteroides* PBio03 and *Leuconostoc mesenteroides* PBio104 for manufacture of commercial kimchi. J. Microbiol. Biotechnol..

[6] Future market insights Inc. 2025. Starter cultures market outlook from 2025 to 2035. https://www.futuremarketinsights.com/reports/starter-cultures-market (Accessed April 8, 2025).

[7] Lee ME, Jang JY, Lee JH, Park HW, Choi HJ, Kim TW (2015). Starter cultures for kimchi fermentation. J. Microbiol. Biotechnol..

[8] Sakai K, Murata Y, Yamazumi H, Tau Y, Mori M, Moriguchi M, Shirai Y (2007). Selective proliferation of lactic acid bacteria and accumulation of lactic acid during open fermentation of kitchen refuse with intermittent pH adjustment. Food Sci. Technol. Res..

[9] Hayek SA, Ibrahim SA (2013). Current limitations and challenges with lactic acid bacteria: a review. Food Nutr. Sci..

[10] Beret MV, Peralta GH, Vera-Candioti L, Wolf IV, Sánchez R, Hynes ER (2021). Culture media based on effluent derived from soy protein concentrate production for *Lacticaseibacillus paracasei* 90 biomass production: statistical optimisation, mineral characterization, and metabolic activities. Antonie Van Leeuwenhoek.

[11] Li X, Chen S, Zhao L, Zeng X, Liu Y, Li C (2023). Effect of lactic acid bacteria by different concentrations of copper based on nontarget metabolomic analysis. Environ. Sci. Pollut. Res..

[12] Blamey J, Chiong M, López C, Smith E (1999). Optimization of the growth conditions of the extremely thermophilic microorganisms *Thermococcus celer* and *Pyrococcus woesei*. J. Microbiol. Methods.

[13] Gao Y, Liang J, Xiao R, Zang P, Zhao Y, Zhang L (2018). Effect of four trace elements on *Paenibacillus polymyxa* Pp-7250 proliferation, activity and colonization in ginseng. AMB Express.

[14] He Y, Chen Z, Liu X, Wang C, Lu W (2014). Influence of trace elements mixture on bacterial diversity and fermentation characteristics of liquid diet fermented with probiotics under air-tight condition. PLoS One.

[15] Sonkar V, Shukla S, Pandey A. 2025. Production optimization of EPS and photosynthetic pigment (chlorophyll-a, chlorophyll-b, carotenoids) production from chlorella using BBD matrix for RSM. *Technologies and Innovations for Sustainable Development*, pp. 134-153, CRC Press.

[16] El-Bendary MA, Afifi SS, Moharam ME, Elsoud MMA, Gawdat NA (2022). Optimization of *Bacillus subtilis* NRC1 growth conditions using response surface methodology for sustainable biosynthesis of gold nanoparticles. Sci. Rep..

[17] Kim N, Park B, Mun SY, Choi EJ, Lee M, Song JH, Kang JY, Kim D, Kim HM (2025). Enhancing neuroinflammatory modulation and sensory qualities in kimchi: a combined starter culture approach. J. Agric. Food Res..

[18] Hwang IM, Yang JS, Jung JH, Lee HW, Lee HM, Seo HY (2019). Dietary intake assessment of macro, trace, and toxic elements via consumption of kimchi in South Korea. J. Sci. Food Agric..

[19] U.S. Food and Drug Administration. 2022. GRAS Substances (SCOGS) Database. https://www.hfpappexternal.fda.gov/scripts/fdcc/index.cfm?set=SCOGS (Accessed June 6, 2025).

[20] Eades  CH, Womack M (1953). Calcium as a growth stimulant for *Lactobacillus casei*. J. Bacteriol..

[21] Lew LC, Liong MT, Gan CY (2013). Growth optimization of *Lactobacillus rhamnosus* FTDC 8313 and the production of putative dermal bioactives in the presence of manganese and magnesium ions. J. Appl. Microbiol..

[22] Christensen DG, Orr JS, Rao CV, Wolfe AJ (2017). Increasing growth yield and decreasing acetylation in *Escherichia coli* by optimizing the carbon-to-magnesium ratio in peptide-based media. Appl. Environ. Microbiol..

[23] Givry S, Duchiron F (2008). Optimization of culture medium and growth conditions for production of L-arabinose isomerase and Dxylose isomerase by *Lactobacillus bifermentans*. Microbiology.

[24] Watanabe M, van der Veen S, Nakajima H, Abee T (2012). Effect of respiration and manganese on oxidative stress resistance of *Lactobacillus plantarum* WCFS1. Microbiology.

[25] Yu AO, Wei L, Marco ML (2022). Calcium determines *Lactiplantibacillus plantarum* intraspecies competitive fitness. Appl. Environ. Microbiol..

[26] Dominguez DC (2004). Calcium signalling in bacteria. Mol. Microbiol..

[27] Sahalan AZ, Abd Aziz AH, Lian HH, Abd Ghani MK (2013). Divalent Cations (Mg^2+^, Ca^2+^) protect bacterial outer membrane damage by polymyxin B. Sains Malays..

[28] Yang Y, Huang S, Wang J, Jan G, Jeantet R, Chen X (2017). Mg^2+^ improves the thermotolerance of probiotic *Lactobacillus rhamnosus* GG, *Lactobacillus casei* Zhang and *Lactobacillus plantarum* P‐8. Lett. Appl. Microbiol..

[29] Helmann JD. 2025. Metals in motion: understanding labile metal pools in bacteria. *Biochemistry* https://doi.org/10.1021/acs.biochem.4c00726. 10.1021/acs.biochem.4c00726 39755956 PMC11755726

[30] Elli M, Zink R, Rytz A, Reniero R, Morelli L (2000). Iron requirement of *Lactobacillus* spp. in completely chemically defined growth media. J. Appl. Microbiol..

[31] Li L, Ma Y (2014). Effects of metal ions on growth, β-oxidation system, and thioesterase activity of *Lactococcus lactis*. J. Dairy Sci..

[32] Osman D, Martini MA, Foster AW, Chen J, Scott AJ, Morton RJ (2019). Bacterial sensors define intracellular free energies for correct enzyme metalation. Nat. Chem. Biol..

[33] Li P, Xie C, Zeng Q, Yuan Y (2023). Effect of different hydrophobic soybean isolated peptides and their zinc complexes on the growth and fermentation of *Lactobacillus bulgaricus*. Int. J. Food Sci. Technol..

[34] Ma Z, Faulkner MJ, Helmann JD (2012). Origins of specificity and cross‐talk in metal ion sensing by *Bacillus subtilis* Fur. Mol. Microbiol..

[35] Gat O, Mendelson I, Chitlaru T, Ariel N, Altboum Z, Levy H (2005). The solute‐binding component of a putative Mn (II) ABC transporter (MntA) is a novel *Bacillus anthracis* virulence determinant. Mol. Microbiol..

[36] Rayman MK, MacLeoD RA (1975). Interaction of Mg^2+^ with peptidoglycan and its relation to the prevention of lysis of a marine pseudomonad. J. Bacteriol..

[37] Tang D, Wu Y, Wu L, Bai Y, Zhou Y, Wang Z (2022). The effects of ammonia stress exposure on protein degradation, immune response, degradation of nitrogen-containing compounds and energy metabolism of Chinese mitten crab. Mol. Biol. Rep..

[38] Long Q, Zhou Q, Ji L, Wu J, Wang W, Xie J (2012). *Mycobacterium smegmatis* genomic characteristics associated with its saprophyte lifestyle. J. Cell. Biochem..

[39] Thomas KJ, Rice CV (2014). Revised model of calcium and magnesium binding to the bacterial cell wall. BioMetals.

[40] Thomas III KJ, Rice CV (2015). Equilibrium binding behavior of magnesium to wall teichoic acid. Biochim. Biophys. Acta-Biomembr..

[41] Green NM, Taylor WR, Brandl C, Korczak B, MacLennan DH. 2007. Structural and mechanistic implications of the amino acid sequence of calcium‐transporting ATPases. *Ciba foundation symposium 122‐calcium and the cell: calcium and the cell: ciba foundation symposium* 122, pp. 93-119, Wiley Online Library. 10.1002/9780470513347.ch7 2947788

[42] Esyunina D, Kulbachinskiy A (2015). Purification and characterization of recombinant *Deinococcus radiodurans* RNA polymerase. Biochem.-Moscow.

[43] Crona M, Torrents E, Røhr ÅK, Hofer A, Furrer E, Tomter AB (2011). NrdH-redoxin protein mediates high enzyme activity in manganese-reconstituted ribonucleotide reductase from *Bacillus anthracis*. J. Biol. Chem..

[44] Yamamoto N, Sampei G, Kawai G (2022). Free-energy profile analysis of the catalytic reaction of glycinamide ribonucleotide synthetase. Life.

[45] Truglio JJ, Croteau DL, Van Houten B, Kisker C (2006). Prokaryotic nucleotide excision repair: the UvrABC system. Chem. Rev..

[46] Ko YH, Hong S, Pedersen PL (1999). Chemical mechanism of ATP synthase: magnesium plays a pivotal role in formation of the transition state where ATP is synthesized from ADP and inorganic phosphate. J. Biol. Chem..

[47] Lee DD, Galera-Laporta L, Bialecka-Fornal M, Moon EC, Shen Z, Briggs SP (2019). Magnesium flux modulates ribosomes to increase bacterial survival. Cell.

[48] Nierhaus KH (2014). Mg^2+^, K^+^, and the ribosome. J. Bacteriol..

[49] Walker GM (1998). Magnesium as a stress-protectant for industrial strains of *Saccharomyces cerevisiae*. J. Am. Soc. Brew. Chem..

[50] Shulga YV, Topham MK, Epand RM (2011). Regulation and functions of diacylglycerol kinases. Chem. Rev..

[51] Paquete-Ferreira J, Freire F, Fernandes HS, Muthukumaran J, Ramos J, Bryton J (2024). Structural insights of an LCP protein-LytR-from *Streptococcus dysgalactiae subs. dysgalactiae* through biophysical and *in silico* methods. Front. Chem..

[52] Liu C, Zhu X, You L, Gin KY-H, Chen H, Chen B (2023). Per/polyfluoroalkyl substances modulate plasmid transfer of antibiotic resistance genes: a balance between oxidative stress and energy support. Water Res..

[53] Tarling EJ, Edwards PA (2011). ATP binding cassette transporter G1 (ABCG1) is an intracellular sterol transporter. Proc. Natl. Acad. Sci. USA.

[54] Fitzpatrick JJ, Ahrens M, Smith S (2001). Effect of manganese on *Lactobacillus casei* fermentation to produce lactic acid from whey permeate. Process Biochem..

[55] Kundumani-Sridharan V, Subramani J, Das KC (2015). Thioredoxin activates MKK4-NFκB pathway in a redox-dependent manner to control manganese superoxide dismutase gene expression in endothelial cells. J. Biol. Chem..

[56] Gray MD, Bagdasarian M, Hol WG, Sandkvist M (2011). *In vivo* cross‐linking of EpsG to EpsL suggests a role for EpsL as an ATPase‐pseudopilin coupling protein in the Type II secretion system of *Vibrio cholerae*. Mol. Microbiol..

[57] Wu Q, Zhang C, Wa Y, Qu H, Gu R, Chen D, Song Z, Chen X (2022). Correlation between exopolysaccharide biosynthesis and gastrointestinal tolerance of *Lactiplantibacillus plantarum*. J. Appl. Microbiol..

[58] Zhang Y, Chen X, Hu P, Liao Q, Luo Y, Li J (2021). Extraction, purification, and antioxidant activity of exopolysaccharides produced by *Lactobacillus kimchi* SR8 from sour meat *in vitro* and *in vivo*. CyTA-J. Food.

[59] Irie Y, Borlee BR, O'Connor JR, Hill PJ, Harwood CS, Wozniak DJ (2012). Self-produced exopolysaccharide is a signal that stimulates biofilm formation in *Pseudomonas aeruginosa*. Proc. Natl. Acad. Sci. USA.

[60] Othman M, Ariff AB, Rios-Solis L, Halim M (2017). Extractive fermentation of lactic acid in lactic acid bacteria cultivation: a review. Front. Microbiol..

[61] Everaert C, Luypaert M, Magg JLV, Cheng QX, Dinger ME, Hellemans J (2017). Benchmarking of RNA-sequencing analysis workflows using whole-transcriptome RT-qPCR expression data. Sci. Rep..

