# Supplementary material for: Optimizing Divalent Cation Supplementation to Enhance the Production of the Kimchi Starter Strain Latilactobacillus curvatus WiKim0094
Source: J Microbiol Biotechnol. 2025 Jul 18;35:e2505011. doi: 10.4014/jmb.2505.05011 (PMC12324997; doi:10.4014/jmb.2505.05011)
Supplement: Supplementary file 1 [file jmb-35-e2505011-supple.pdf]

J. Microbiol. Biotechnol. 2025. 35: e2505011
https://doi.org/10.4014/jmb.2505.05011

Supplementary Table and Figures

Table S1. Primers used this study and PCR product size isolated from *Latilactobacillus curvatus* WiKim0094.

Gene	Primer nucleotide sequence		Gene size (bp)
WK_00154	Forward	GGA AGC GTT AGC ACC TGA AG	225
	Reverse	GGG ATT GCC CCA TTA TCT TT	
WK_00320	Forward	ACA CCC AAA GGG ATT GAT GA	184
	Reverse	TTT CAA ACC CGT GCA CAA TA	
WK_00362	Forward	AAC TGG GGC CTC GTT AAA GT	155
	Reverse	TCA CCC ATG ACA ACG ACT GT	
WK_00392	Forward	GAA TAT CTC GTG CCC AAA CG	247
	Reverse	GAG AAT TGG TTG GCG TGT TT	
WK_00394	Forward	TTA TCC AAA AGT CCG CAA GC	180
	Reverse	TGA CCG TGA TCT TTC GGT TT	
WK_00752	Forward	TAT GTC GGC GTT GAT GGT AA	238
	Reverse	GCA TTT CTT TGC CAG TTG GT	
WK_00935	Forward	GGT TAT CAA TCG GGT TGT GG	200
	Reverse	CTT CGT CAA CAG TGG CTT GA	
WK_00967	Forward	GGT GAA AGC CGC TTA TTC AA	223
	Reverse	GTA AAC CCC TTG TGC TTG GA	
recA	Forward	TGG GGA ACA AGG CTT AGA AA	152
	Reverse	ACA TCA AGC GGG CTT GTA AC	
gyrB	Forward	GCG GGT CAA TAT AAC GAT GG	158
	Reverse	TCC CGT TCT TTG AGC AAG TT	
rpoB	Forward	GGA TGC TTT GGC ACC ATA CT	170
	Reverse	TGG CAT CGA TAT GAC CGT TA	

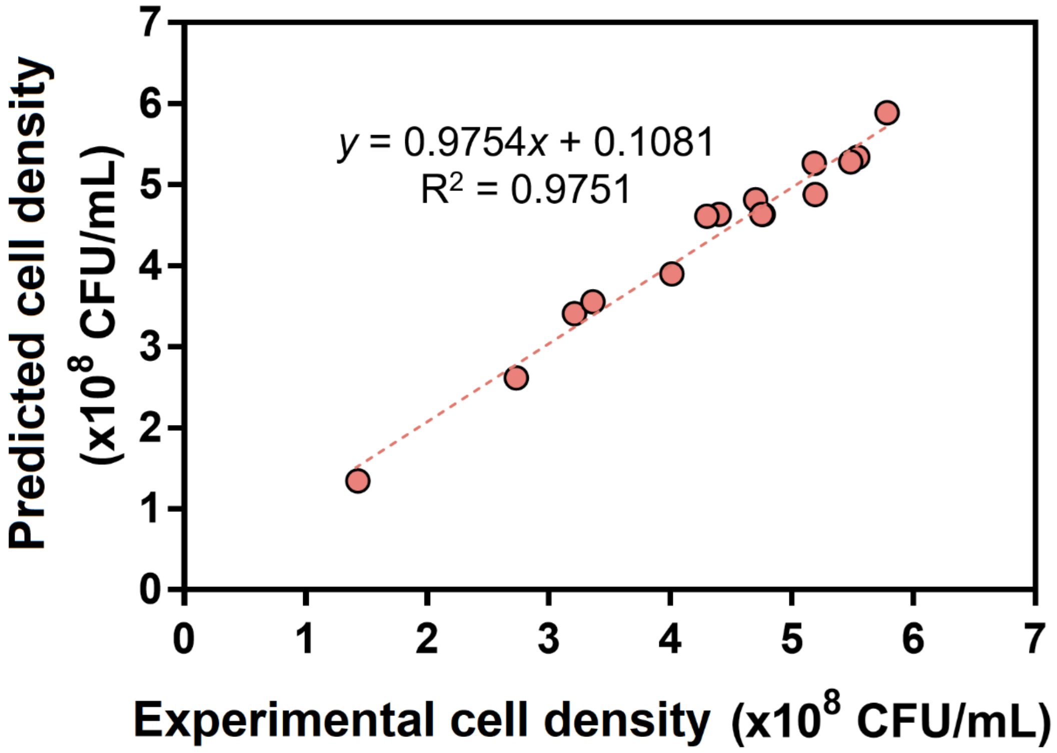

Fig. S1. Correlation between measured and predicted colony-forming units of *L. curvatus* WiKim0094 based on the response surface model.

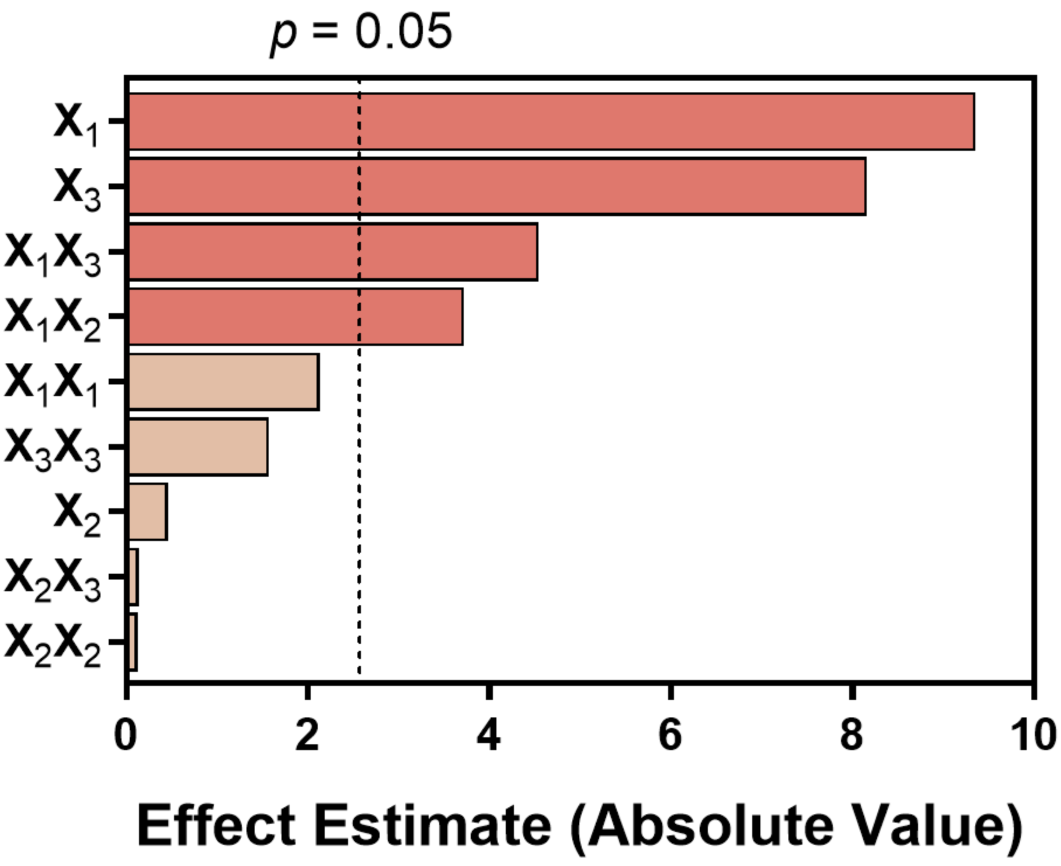

Fig. 2. Pareto chart showing the influence of independent variables on viable cell count in *L. curvatus* WiKim0094. Independent variables: X_1, $MgSO_4$; X_2, $MnSO_4$; X_3, $CaCl_2$.
